# When “Pneumonia” Does Not Resolve: A Case of Pulmonary Embolism Leading to Cavitary Pulmonary Infarction

**DOI:** 10.7759/cureus.107247

**Published:** 2026-04-17

**Authors:** Aryan Neupane, Anna Diamante, Ashmita Chhetri, Evans Kyei-Nimako, Abdelmohaymin Abdalla

**Affiliations:** 1 Internal Medicine, North Alabama Medical Center, Florence, USA; 2 Pulmonary and Critical Care Medicine, North Alabama Medical Center, Florence, USA

**Keywords:** cavitary lung lesion, community-acquired pneumonia (cap), heart failure, necrotizing pneumonia, pulmonary embolism, pulmonary infarction

## Abstract

Pulmonary infarction (PI) is a recognized but relatively uncommon complication of pulmonary embolism (PE). Because its clinical presentation and imaging findings often overlap with those of community-acquired pneumonia (CAP), PI is frequently misdiagnosed. Progression to cavitation is exceedingly rare and typically reflects extensive, irreversible parenchymal necrosis.

We describe a 66-year-old woman with heart failure with reduced ejection fraction (HFrEF) and coronary artery disease (CAD) who initially presented with symptoms consistent with CAP. Although she initially improved, she re-presented ten days later with acute hypoxemic respiratory failure and a newly developed smooth-walled cavitary lung lesion. Chest computed tomography confirmed a submassive PE complicated by necrotizing PI and secondary abscess formation. Given the failure of conservative management and evidence of parenchymal gangrene with the need for source control, she underwent urgent right lateral thoracotomy with bilobectomy for source control. The post-operative course was complicated by refractory shock and pulseless electrical activity (PEA) arrest. Following goals-of-care discussions, the family elected to pursue comfort-focused care.

This case shows the importance of systemic-to-pulmonary collateral circulation in preserving lung parenchymal viability. In patients with limited cardiovascular reserve, failure of bronchial arterial compensation can precipitate catastrophic necrotizing infarction. Clinicians should maintain a high index of suspicion for PI in cases of “nonresolving pneumonia,” as early recognition may be critical in preventing progression to cavitary disease.

## Introduction

Pulmonary infarction (PI) is a known but uncommon complication of pulmonary embolism (PE), occurring in approximately 10%-15% of cases, and is frequently misdiagnosed as community-acquired pneumonia (CAP) because of overlapping clinical and radiographic features [1]. Progression to cavitation is an exceedingly rare phenomenon, occurring in approximately 4%-7% of PI cases, and is associated with significant morbidity and mortality [2]. We report a rare case of PE presenting as pneumonia, with subsequent progression to necrotizing PI and abscess formation.

## Case presentation

**Figure 1:**
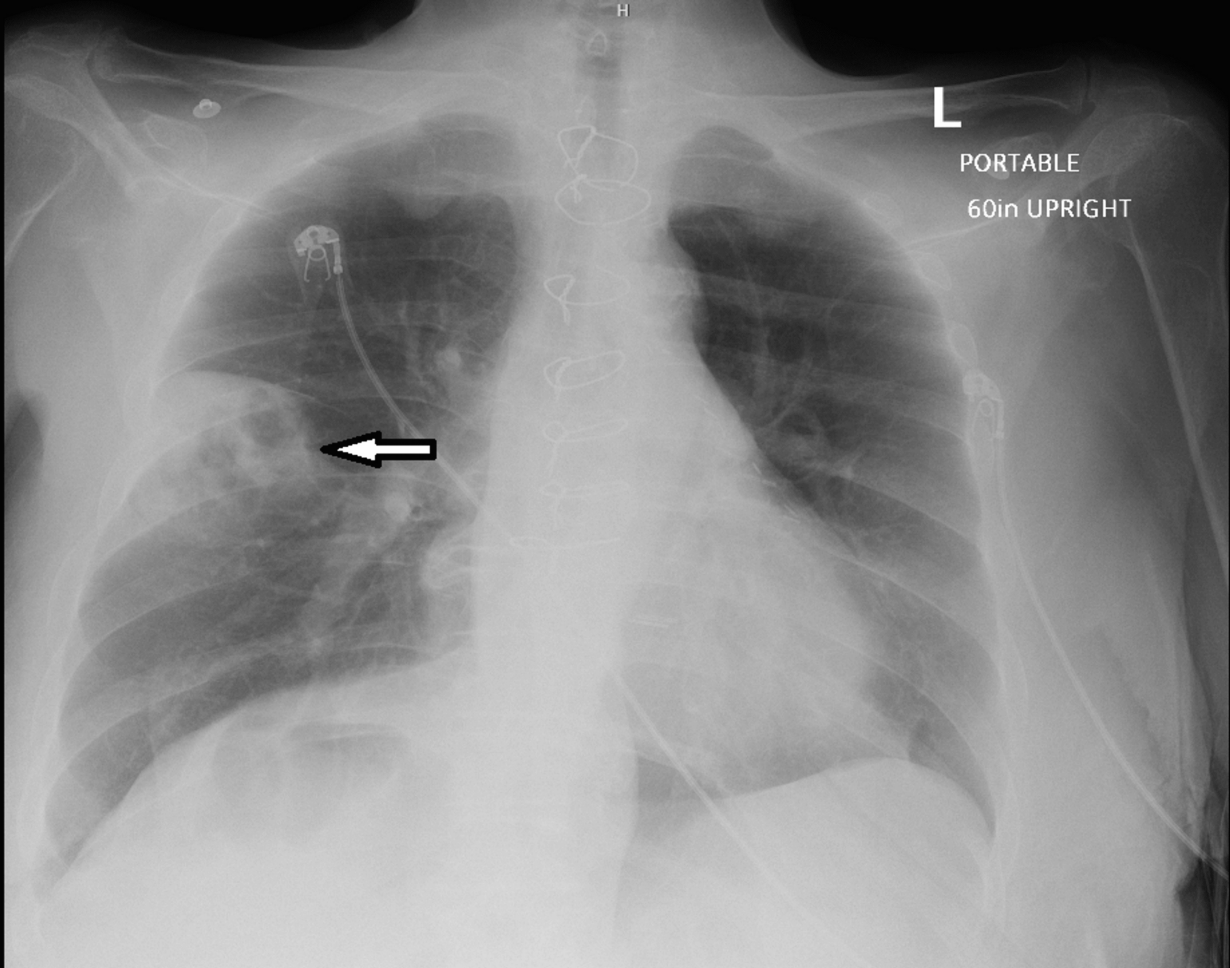
Chest radiograph obtained at initial admission demonstrating right middle lobe opacity.

A 66-year-old female with a medical history of CAD status post coronary artery bypass grafting (CABG), paroxysmal atrial fibrillation with nonadherence to apixaban, HFrEF, and type 2 diabetes presented with fever, cough, palpitations, and generalized weakness. Physical examination revealed mild tachypnea and tachycardia, and laboratory workup was notable for leukocytosis of 14 × 10³/µL (reference range, 4-11 × 10³/µL) (Table 1). A chest radiograph (CXR) demonstrated a right mid-lung consolidation (Figure 1). Given the clinical constellation of fever, cough, and radiographic findings, she was diagnosed with CAP, treated with intravenous levofloxacin, and discharged on a course of oral levofloxacin following clinical improvement.

**Table 1:** Pertinent laboratory findings at readmission.

Laboratory Test	Result	Reference Range
Leukocyte count	14,000/mm³	4,300-11,000/mm³
Platelet count	335 × 10³/mm³	150-375 × 10³/mm³
Hemoglobin	14 g/dL	12-16 g/dL
Serum lactate	5.2 mmol/L	0.7-1.9 mmol/L

**Figure 2:**
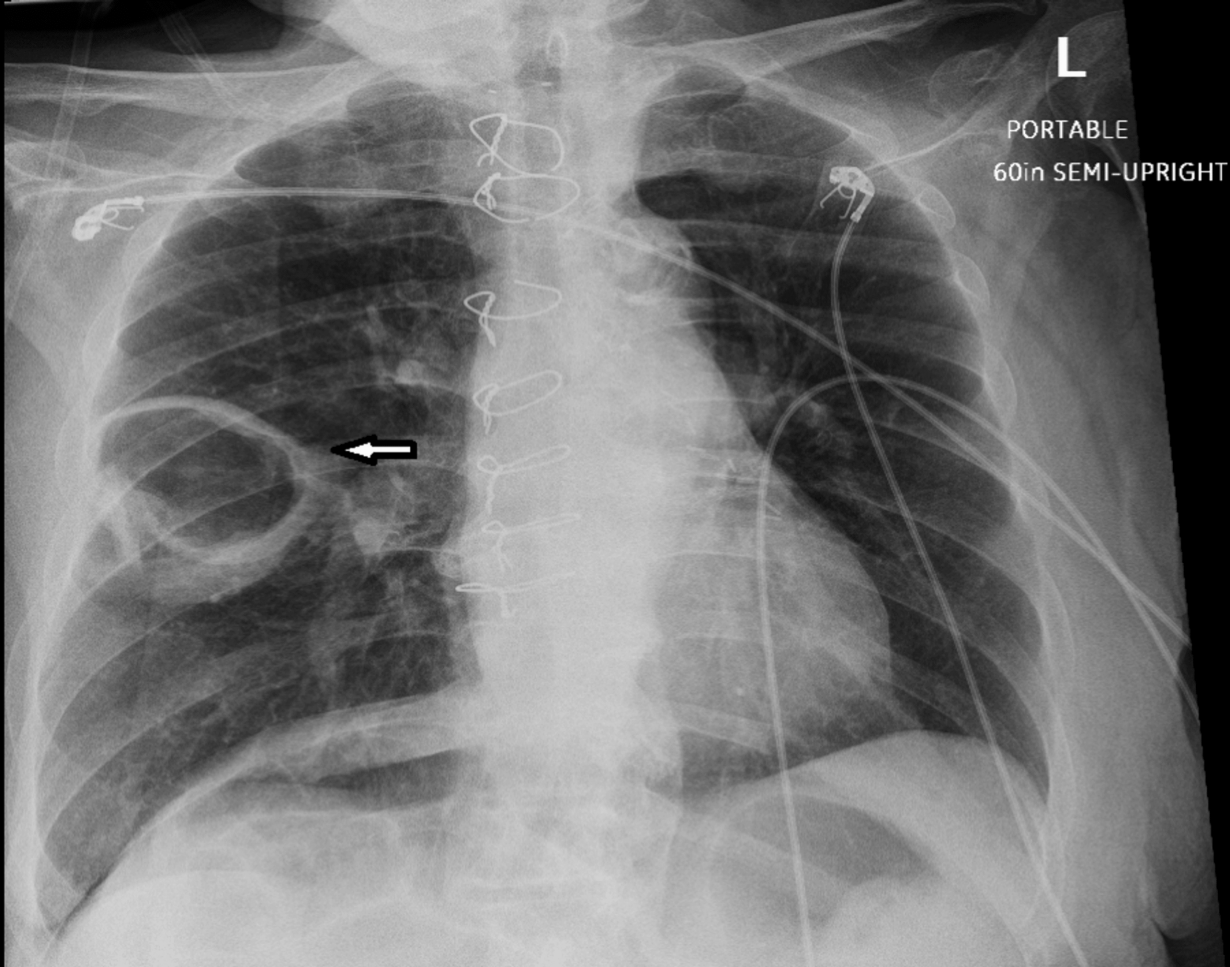
Chest radiograph obtained at subsequent admission demonstrating a new smooth-walled cavitary lesion in the right middle lobe, in the prior area of consolidation.

**Figure 3:**
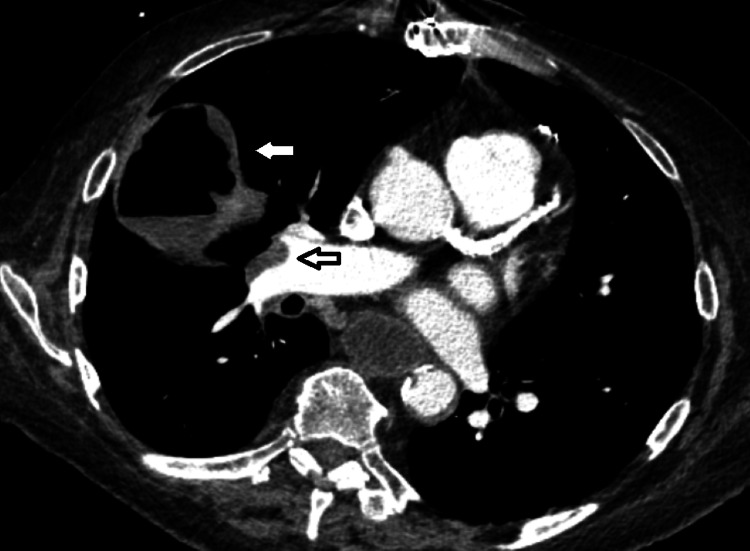
Mediastinal window CT images demonstrating a cavitary lesion with an air–fluid level in the right middle lobe abutting the pleura (white arrow) and partial visualization of a pulmonary embolus within the right pulmonary artery (black arrow).

**Figure 4:**
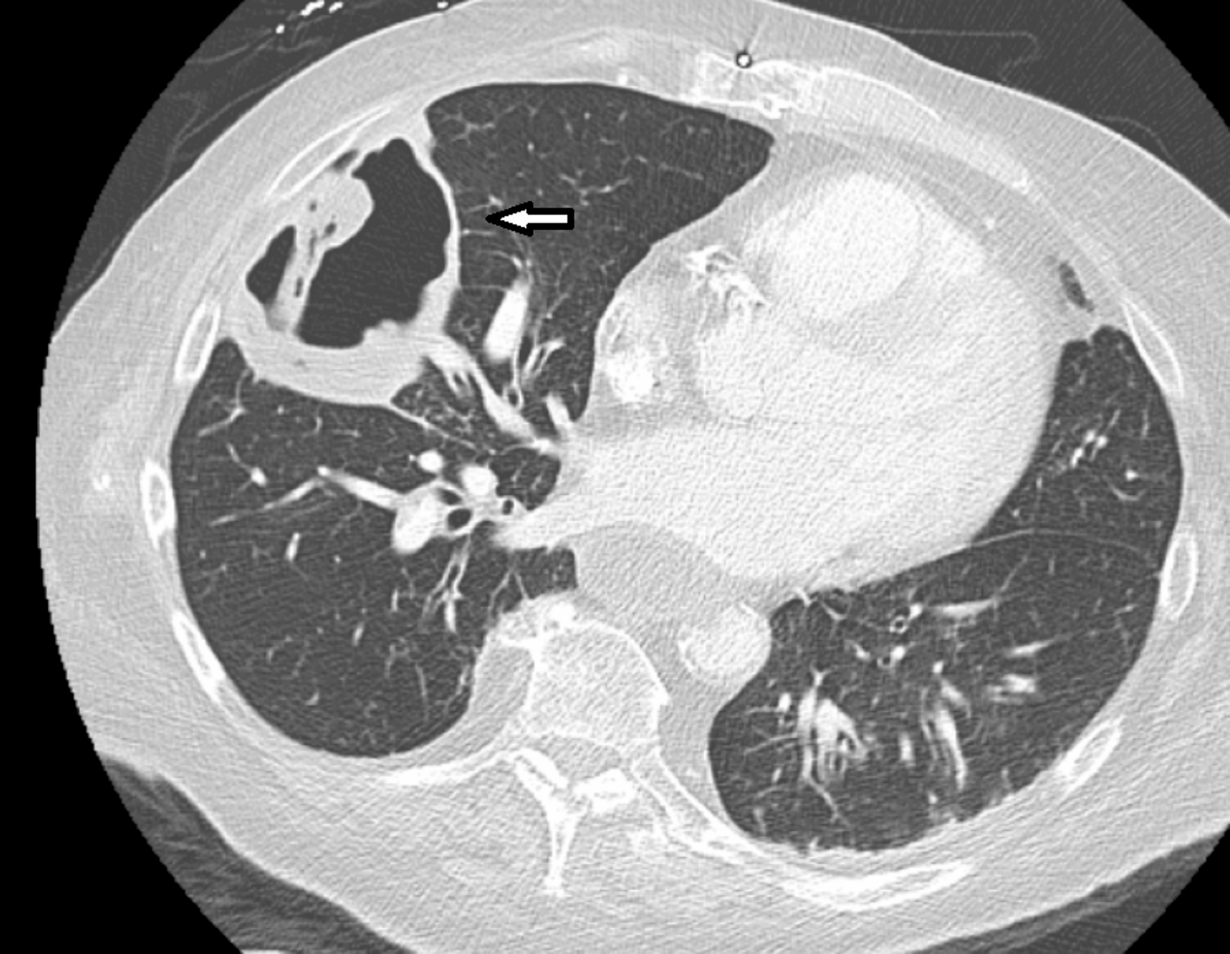
Lung window CT images demonstrating a cavitary lesion with an air–fluid level in the right middle lobe abutting the pleura.

Ten days post-discharge, the patient re-presented with rapid functional decline and worsening cough. She was tachycardic, with a heart rate of 125; hypoxic, with an oxygen saturation of 85% on 3 L of supplemental oxygen via nasal cannula; and had an elevated serum lactate of 5.2 mmol/L (reference range, 0.7-1.9 mmol/L). Repeat CXR revealed a newly developed, smooth-walled cavitary lesion in the right mid-lung (Figure 2). Given the rapid progression and new cavitation on CXR, urgent contrast-enhanced chest CT was performed, revealing a partially occluding embolus in the right main pulmonary artery with nonocclusive extension into the right lower lobe pulmonary artery. Imaging also demonstrated a cavitary lesion with an air-fluid level in the right middle lobe abutting the pleura (Figures 3-4), findings most consistent with pulmonary infarction complicated by necrosis and superimposed abscess formation. There were no signs of right ventricular strain on echocardiography. The patient was initially managed with therapeutic anticoagulation using heparin and broad-spectrum intravenous antibiotics, including vancomycin and meropenem.

With no improvement in clinical status, the patient underwent a right lateral thoracotomy with bilobectomy of the right upper and middle lobes for source control. Intraoperative findings revealed a large abscess cavity within the right upper lobe with extension into the right middle lobe. Although she was initially extubated on postoperative day one, her course was complicated by sudden hemodynamic collapse and pulseless electrical activity (PEA) arrest. Despite appropriate resuscitative efforts, the patient developed refractory shock. Following goals-of-care discussions, the family elected to pursue comfort-focused care. The patient died shortly thereafter. 

## Discussion

This case serves as a quintessential example of PI as a “great mimicker.” The diagnostic anchor of pneumonia is easily set because PI and CAP share a near-identical clinical profile, including fever, cough, leukocytosis, and pleuritic pain [3,4]. In this patient, the initial right mid-lung consolidation was managed as infectious CAP; however, in retrospect, this likely represented “Hampton’s hump”, a pleural-based wedge opacity signifying PI that is frequently indistinguishable from an infectious process in its initial stages [5,6].

The progression from an initially isolated embolic event to fatal lung necrosis reflects failure of the lung’s dual blood supply. Normally, the bronchial arterial circulation serves as an effective collateral network, helping maintain tissue perfusion when pulmonary arterial flow is obstructed. In patients with HFrEF and CAD, limited cardiovascular reserve may compromise this compensatory mechanism, leaving the lung parenchyma more vulnerable to ischemia [7,8]. As a result, what is typically a survivable thromboembolic event can evolve into a devastating ischemic process.

In this case, the pulmonary consolidation progressed to cavitation over approximately 10 days, a timeline consistent with necrotizing PI. As infarcted lung tissue undergoes necrosis, it becomes susceptible to secondary bacterial infection, evolving from an initially sterile ischemic injury into a septic pulmonary abscess. This process significantly complicates management, as impaired perfusion may limit effective antibiotic penetration into the necrotic core [9].

Rapid progression of a presumed pneumonic consolidation into a cavitary lesion should prompt reconsideration of an infectious diagnosis and raise concern for an underlying vascular process. Although surgical intervention in this case aimed to achieve source control of necrotic lung tissue [10], the patient’s ongoing thrombotic risk and limited cardiac reserve highlight the high mortality associated with cavitary PI.

## Conclusions

PI can closely mimic CAP, as both conditions often present with similar symptoms and radiographic findings, making early distinction challenging. For this reason, a high index of suspicion is especially important in patients with significant cardiovascular risk factors. In those with HFrEF and underlying coronary artery disease, impaired bronchial arterial collateral perfusion may limit compensatory blood flow, increasing vulnerability to lung necrosis following pulmonary embolism. The subsequent appearance of a cavitary lung lesion within about 1-2 weeks after an initial diagnosis of “pneumonia” follows a recognized temporal pattern of necrotizing PI and should prompt clinicians to reconsider the original diagnosis.

## References

[1] Okwudire EG, Ezenwugo UM, Ugwoegbu JU, Isiozor I, Ofeimun VE, Obiagwu UU (2022). Cavitary pulmonary infarction mimicking Koch’s disease. Sci Res.

[2] Libby LS, King TE, LaForce FM, Schwarz MI (1985). Pulmonary cavitation following pulmonary infarction. Medicine (Baltimore).

[3] Black AD (2016). Non-infectious mimics of community-acquired pneumonia. Pneumonia (Nathan).

[4] Nilgiri K M, Orakkan RG, Meshram SB (2024). Cavitary consolidation in pulmonary infarct secondary to embolism: a rare presentation. Cureus.

[5] Kubiak K, Bazylewicz-Zakrzewska K, Poncyljusz W (2025). Hampton's hump—a rare radiological feature in patients with pulmonary embolism in a single-center study. J Clin Med.

[6] Zhang HP, Wu XH, Zhao TR (2022). CT manifestations of pulmonary infarction secondary to acute pulmonary embolism. CT Theory Appl.

[7] Tsao MS, Schraufnagel D, Wang NS (1982). Pathogenesis of pulmonary infarction. Am J Med.

[8] Libby P, Bonow RO, Mann DL, Tomaselli GF, Bhatt DL, Solomon SD (2021). Braunwald's Heart Disease: A Textbook of Cardiovascular Medicine.

[9] Koroscil MT, Hauser TR (2017). Acute pulmonary embolism leading to cavitation and large pulmonary abscess: a rare complication of pulmonary infarction. Respir Med Case Rep.

[10] Reimel BA, Krishnadasen B, Cuschieri J, Klein MB, Gross J, Karmy-Jones R (2006). Surgical management of acute necrotizing lung infections. Can Respir J.

